# Deciphering the Morphology Change and Performance Enhancement for Perovskite Solar Cells Induced by Surface Modification

**DOI:** 10.1002/advs.202205342

**Published:** 2022-12-01

**Authors:** Nianci Guan, Yuezhou Zhang, Wei Chen, Zhengyan Jiang, Lei Gu, Ruixue Zhu, Deependra Yadav, Deli Li, Baomin Xu, Leifeng Cao, Xingyu Gao, Yonghua Chen, Lin Song

**Affiliations:** ^1^ Frontiers Science Center for Flexible Electronics Institute of Flexible Electronics (IFE) Northwestern Polytechnical University (NPU) Xi'an 710072 P. R. China; ^2^ Shenzhen Key Laboratory of Ultraintense Laser and Advanced Material Technology, Center for Advanced Material Diagnostic Technology, and College of EngineeringPhysics Shenzhen Technology University (SZTU) Lantian Road 3002 Shenzhen 518118 China; ^3^ Department of Materials Science and Engineering Southern University of Science and Technology Shenzhen 518055 China; ^4^ Pharmaceutical Sciences Laboratory Faculty of Science and Engineering Åbo Akademi University Turku FI‐00520 Finland; ^5^ Fujian Cross Strait Institute of Flexible Electronics (Future Technologies) Fujian Normal University Fuzhou 350117 China; ^6^ Country Shanghai Synchrotron Radiation Facility (SSRF) Zhangjiang Lab Shanghai Advanced Research Institute Chinese Academy of Sciences Shanghai 201204 China; ^7^ Key Laboratory of Flexible Electronics (KLOFE) and Institute of Advanced Materials (IAM) Jiangsu National Synergistic Innovation Center for Advanced Materials (SICAM) Nanjing Tech University (NanjingTech) 30 South Puzhu Road Nanjing Jiangsu 211816 China

**Keywords:** BTACl modification, device physics, low dimensional perovskites, morphology, perovskite solar cells

## Abstract

Organic–inorganic perovskite solar cells (PSCs) have achieved great attention due to their expressive power conversion efficiency (PCE) up to 25.7%. To improve the photovoltaic performance of PSCs, interface engineering between the perovskite and hole transport layer (HTL) is a widely used strategy. Following this concept, benzyl trimethyl ammonium chlorides (BTACls) are used to modify the wet chemical processed perovskite film in this work. The BTACl‐induced low dimensional perovskite is found to have a bilayer structure, which efficiently decreases the trap density and improves the energy level alignment at the perovskite/HTL interface. As a result, the BTACl‐modified PSCs show an improved PCE compared to the control devices. From device modeling, the reduced charge carrier recombination and promoted charge carrier transfer at the perovskite/HTL interface are the cause of the open‐circuit (*V*_oc_) and fill factor (FF) improvement, respectively. This study gives a deep understanding for surface modification of perovskite films from a perspective of the morphology and the function of enhancing photovoltaic performance.

## Graphical abstract

A pure 2D perovskite and a 2D/3D perovskite mixture are formed on the MAPbI_3_ film after surface treatments. This bilayer structure gives rise to a reduced trap density and an improved energy level alignment between the perovskite and HTL. Via simulation, these two positive effects are verified to be the cause of *V*_oc_ and fill factor (FF) improvement, respectively.

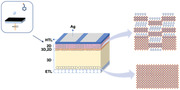

## 1 Introduction

Organic–inorganic perovskite solar cells (PSCs) have been the focus of an increasing research interest in emerging photovoltaics as their power conversion efficiency (PCE) has reached a milestone of over 25% in just a dozen years.^[^ ^1^, ^2^ ^]^ Compared with other solar cells like monocrystalline silicon and thin film cells, PSCs have unique merits of tunable bandgap, strong light absorption, long exciton diffusion, and high charge mobility. Over the past decade, due to the advances in production routines such as the development of low‐cost and large‐scale manufacturing technologies, PSCs hold a great promise for commercialization.

For three‐dimensional (3D) bulk perovskites, the film quality can be enhanced by crystallinity tailoring and composition engineering, which is expected to improve the photovoltaic performance of PSCs.^[^ ^3^, ^4^, ^5^ ^]^ For example, Gao et al. employed imidazole bromide functionalized graphene quantum dots (I‐GQDs) to optimize the interface between SnO_2_ and perovskite.^[^ ^5^, ^6^ ^]^ The functional groups gave rise to an increased perovskite grain size and an improved film quality, which reduced the interface defect densities. As a result, a champion PCE of 22.37% with distinguished stability was obtained for the modified device.^[^ ^7^ ^]^ Wang et al. proposed a strategy of using guanidine ions (Gua^+^) to regulate methylammonium (MA)/formamidinium (FA) based perovskite composition for defect passivation and reduction of nonradiative‐recombination.^[^ ^8^ ^]^ Moreover, by introducing large A‐site cations into perovskites, the 3D structure could be transformed to 2D form. Due to the excellent stability and remarkable moisture‐resistant ability, 2D perovskites as a passivating layer over 3D counterparts have been developed to improve the PCE and operational stability of PSCs. For example, Cho et al. introduced a 2D perovskite thin layer with a wide band gap on top of the 3D perovskite film, which led to an improved PCE. Additionally, the PSC with this layer‐by‐layer 2D/3D hybrid structure was found to be more stable than the device based on pure 3D perovskites.^[^ ^9^ ^]^ However, these methods have a limited effect on the improvement of the interface between the perovskite layer and the hole transport layer (HTL), which plays a significant role in charge‐carrier transfer from the perovskite to HTL.^[^ ^10^ ^]^ A poor contact leads to additional nonradiative recombination, which results in a low open‐circuit voltage (*V*_oc_). To address this problem, modification of perovskite film surface with trimethyl ammonium salts is widely used to optimize the perovskite/HTL contact. For example, Jung et al. employed *n*‐hexyl trimethyl ammonium bromides to intercalate a layer of wide‐bandgap perovskite at the perovskite/HTL interface.^[^ ^11^, ^12^ ^]^ This strategy led to a dramatic improvement of *V*_oc_, from 0.92 eV for the pristine PSC to 1.14 eV for the treated cell.^[^ ^12^, ^13^ ^]^ Wang et al. proposed the use of cetyl trimethyl ammonium bromide (CTAB) for interface modification between the perovskite and HTL. It was found that the CTA^+^ anchored to the perovskite film to passivate surface defects and Br^−^ was gradient distributed on the film surface.^[^ ^14^ ^]^ This occurrence resulted in a PCE enhancement for the modified PSCs.

In this work, we develop a trimethyl ammonium salt, benzyl trimethyl ammonium chloride (BTACl), to treat the surface of perovskite films for efficient photovoltaic devices. On the one hand, the functionalized moiety (−N^+^(CH_3_)_3_) allows BTACl molecules to react with the prepared perovskite to form a capping layer. On the other hand, the aromatic moiety is expected to improve interfacial contact between perovskite and spiro‐OMeTAD via favorable van der Waals interactions. Interestingly, a bilayer consisting of pure 2D (BTA)_2_MAPbI_4_ and 2D/3D mixed perovskites is formed on top of perovskite films rather than a 2D perovskite monolayer, which is observed for the first time. This structure promotes a better energy level alignment between the active layer and HTL compared to pure 2D perovskite layers, results in more efficient charge carrier transfer. Moreover, a significantly reduced trap density at the film surface is observed, leading to a more efficient charge carrier transport and extraction. Consequently, the BTACl‐treated devices exhibit a champion efficiency of 21.72%, displaying a 18% enhancement compared to the control PSC (a champion efficiency of 18.43%). Furthermore, the BTACl‐treated PSCs maintain 90% of their initial PCE more than 1000 h, whereas the pristine PSCs show 34% loss in PCE under the same aging condition. The mechanism study in this work gives a detailed information about the film structure after BTACl treatments, which lays the foundation for unraveling the correlation between the morphology optimization and performance enhancement.

## 2 Results and Discussion

**Figure 1:**
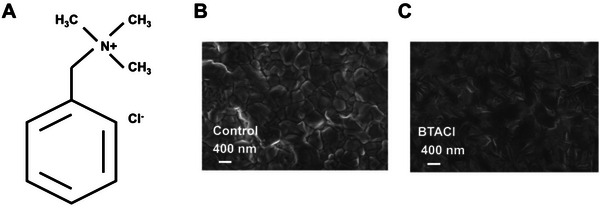
A) Chemical formula of the benzyl trimethyl ammonium chlorides (BTACl) molecular. Scanning electron microscope (SEM) images of perovskite films (B) without and (C) with the BTACl modification.

**Figure** **1**A depicts the molecular structure of BTACl, which is employed to modify the perovskite‐film surface. The size of this molecule is calculated to be 6.74 Å (Figure S1, Supporting Information). To study the BTACl treatment on the morphology of perovskite films, the surface nanostructures of perovskite films before and after BTACl modification are firstly probed with plan view scanning electron microscope (SEM) measurements (Figure 1B,C).^[^ ^15^ ^]^ Both samples display a flat surface without pinholes, which is favorable for photovoltaic applications. However, the crystal size differs. The control film shows a close‐packed array of perovskite crystals with an average size of (249 ± 87) nm, and the crystal boundaries are observed very clearly (Figure 1B). After the BTACl modification, larger crystal size and blurred crystal boundaries are obtained (Figure 1C). The morphology change is might due to the formation of 2D perovskites on top of the film, as similar observations were frequently reported in literature.^[^ ^16^ ^]^ In addition, the morphology difference is confirmed with atomic force microscope (AFM) images, which additionally demonstrate that the root‐mean‐square (RMS) reduces from 60.0 nm for the control sample to 47.2 nm for the film treated with BTACl (Figure S2, Supporting Information). A lower surface roughness is more conducive to contact with organic hole transport materials (HTMs), which is expected to decrease the resistance and facilitate the charge transfer at the interface.

**Figure 2:**
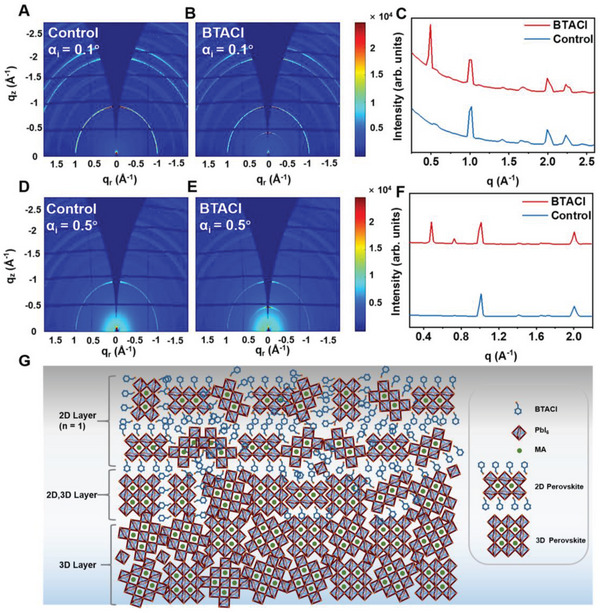
2D GIWAXS data of the perovskite film surfaces A) without and B) with benzyl trimethyl ammonium chloride (BTACl) treatment. C) Vertical sector integrals obtained from (A) and (B). 2D GIWAXS data of the bulk films D) without and E) with BTACl treatment. F) Vertical sector integrals obtained from (D) and (E). G) Schematic illustration of the morphology of the perovskite overlayer induced by the BTACl treatment.

To verify the formation of 2D perovskites on top of the film after BTACl modification, grazing incidence wide‐angle X‐ray scattering (GIWAXS) measurements are performed. By varying incident angles (*α*_i_), this advanced scattering technique allows to probe the surface or inner morphology of the sample independently. In detail, when the *α*_i_ is smaller than the critical angle (*α*_c_) of the probed materials, a total external reflection of X‐rays occurs, thus the penetration depth of the X‐ray is just a few nanometers. As a result, a high sensitivity to surface structures is achieved. For the GIWAXS measurements in this work, the wavelength of the used X‐ray is 1.24 Å. According to the equation $\alpha_{c}=\lambda \sqrt{\frac{\rho }{\pi }}$ (*ρ* is the scattering length density (SLD) of the probed sample), the *α*_c_ of MAPbI_3_ materials is calculated to 0.22°.^[^ ^17^ ^]^ Therefore, we choose a small incident angle of 0.1° to characterize the surface structures of the films in this work. At this incident angle, the penetration depth of the used X‐ray (*Λ*) is determined to be about 5.9 nm based on the equation $\Lambda =\lambda /[\sqrt{2}\pi \left(I_{i}+I_{j}\right)]$ (where $I_{i,j}={\left[\sin^{2}\alpha_{c}-\sin^{2}\alpha_{i,j}+\sqrt{{\left(\sin^{2}\alpha_{c}-\sin^{2}\alpha_{i,j}\right)}^{2}-{\left(2\beta \right)}^{2}}\right]}^{\frac{1}{2}}$, *β* is the absorptive aspect of the wave‐matter interaction).^[^ ^18^, ^19^ ^]^ **Figure** **2**A,B show the 2D GIWAXS data of the perovskite film surfaces before and after BTACl modification, respectively. The observed Debye–Scherrer rings in both samples are identified as the characteristic features of the MAPbI_3_ crystals, suggesting that the BTACl treatment does not alter the crystal phase at the sample surface. Compared to the control sample, an extra Bragg diffraction ring located at *q*_r_ = 0.48 Å^−1^ is present in Figure 2B, which is an indicator of the formation of 2D perovskite structures after BTACl introduction. Cross‐sectional SEM measurements confirm a 2D perovskite layer at the film surface, in which its thickness is determined to be about 19.1 nm (Figure S3, Supporting Information). This value is larger than the X‐ray penetration depth, indicating that the scattering signals contain only the information about the 2D structures when the incident angle of 0.1° is selected in this work.

Figure 2C shows sector‐averaged integrals of the 2D GIWAXS data. It can be seen that the diffraction peak of 2D perovskites is located at 0.48 Å^−1^, which corresponds to a real space distance of 13.08 Å. This value is the sum of the BTACl‐molecule size (6.74 Å) and the lattice constant of cubic MAPbI_3_ (6.28 Å), indicating the presence of *n* = 1 phase. Moreover, diffraction originating from the (110) plane of MAPbI_3_ crystals decreases in intensity for the BTACl‐treated sample as compared to the control sample, which is the result of the formation of 2D perovskites. To qualitatively analyze whether the BTACl treatment has an impact on crystal orientation, the angular information on the (110) plane, tube cuts with an angular width of *q*_r_ = 0.95 − 1.05 Å^−1^ are performed on 2D GIWAXS data and fitted with Gaussian functions (Figure S4, Supporting Information).^[^ ^20^ ^]^ By assuming up all the intensity from isotropic and orientated areas, we calculated that about 21.4% and 25.1% crystals have a normal orientation of the (110) plane for the control and BTACl‐treated sample, respectively.^[^ ^20^ ^]^ This finding suggests that the BTACl treatment almost does not alter the preferred orientation of crystals even after the formation of 2D perovskites at the sample surface.

At a *α*_i_ larger than *α*_c_, X‐ray is able to penetrate the film completely and thus the information about the volume morphology is yielded. In this case, we select an *α*_i_ of 0.5°, which is above the *α*_c_ of MAPbI_3_. The obtained 2D GIWAXS data for both samples (before and after BTACl modification) are displayed in Figure 2D,E. The characteristic Debye–Scherrer rings suggest the presence of MAPbI_3_ in the entire film as well. Sector‐averaged integrals of both 2D GIWAXS data are obtained to quantify the inner film morphology (Figure 2F). For the BTACl treated sample, except for the *n* = 1 phase, no other characteristic peaks of 2D perovskite are observed, indicating that the 19.1‐nm‐thick 2D layer is composed of only *n* = 1 phase. Moreover, it is calculated that about 43.32% of the 2D crystals features a preferred spatial arrangement (normal to the substrate). Moreover, it is noticed that an extra peak from 2D perovskites is located at 0.72 Å^−1^, in which the interplanar spacing is calculated to be 8.72 Å. This value is between the characteristic values of *n* = 1 phase and 3D perovskite lattice constant, so it is concluded that the diffraction peak originates from the mixture of 2D and 3D perovskites. Via calculation, 2D structure is determined to be 35.9 vol% and 3D one accounts for 64.1 vol%. Furthermore, the integrated intensity of the *n* = 1 phase characteristic peak (relative peak area under the curve versus *q* = 0.47 Å^−1^) increases from 13 380 to 25 342 a.u.. Noted that the thickness of the 2D perovskite is 5.9 nm under the *α*_i_ of 0.1°, and then the corresponding thickness is determined to be 12.5 nm under the *α*_i_ of 0.5° as the intensity is linearly correlated to the *Λ*. In order to check the component of the 12.5‐nm‐thick 2D perovskite film, GIWAXS measurements with an *α*_i_ of 0.20° are performed (Figure S5A, Supporting Information). Within this *α*_i_, the *Λ* is just 12.5 nm. Figure S5A (Supporting Information) shows the absence of the diffraction peak located at 0.72 Å^−1^, indicating the 12.5‐nm‐thick 2D film only includes the *n* = 1 phase. With a slight increase of *α*_i_ to 0.21° (*Λ* corresponds to 17.3 nm), the diffraction peak of 2D/3D mixture is observed in GIWAXS data (Figure S5B, Supporting Information). Therefore, the morphology of the perovskite overlayer (Figure S3, Supporting Information) is deciphered, which is a bilayer configuration. The *n* = 1 phase and 2D/3D mixture constitute the top (12.5 nm) and bottom (6.6 nm) parts, respectively. Based on the GIWAXS results, thus, a schematic illustration of this perovskite overlayer is displayed in Figure 2G.

**Figure 3:**
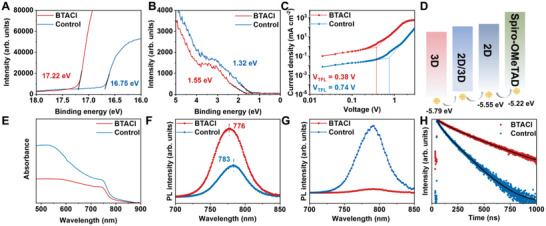
A,B) Ultraviolet photoelectron spectroscopy (UPS) spectra for the perovskitefilms without and with BTACl modification. C) SCLC measurements based on the hole‐only devices without and with the BTACl treatments. D) The energy band diagram of perovskites and Spiro‐OMeTAD. E) UV–vis spectra. PL spectra of the control and BTACl‐treated perovskite films with implemented layouts of F) glass/perovskite and G) glass/perovskite/Spiro‐OMeTAD. H) TRPL spectra of the perovskite films with and without BTACl treatments. The samples are on glass/ITO/SnO_2_ substrates.

In order to study the effect of the BTACl modification on the energy band structure, ultraviolet photoelectron spectroscopy (UPS) measurements are performed on the perovskite films. From the UPS spectra of the control sample (**Figure** **3**A), the work function (*W*_F_) is calculated to be 4.47 eV, which is in line with the value reported in literature.^[^ ^21^ ^]^ After BTACl treatment, the *W*_F_ decreases to 4.0 eV. This upshift of *W*_F_ is frequently observed in literature when perovskites change from 3D to 2D.^[^ ^22^, ^23^, ^24^ ^]^ The gaps between the valence band maxima (VBM) and Fermi level are calculated to be 1.32 and 1.55 eV for the control and BTACl‐treated perovskite films, respectively (Figure 3B). An upshift of the energy level is found in the 2D perovskite compared to the 3D counterpart.^[^ ^25^ ^]^ As UPS measurement is surface sensitive, the energy band structure of the buried 2D/3D mixture is not measurable.^[^ ^26^, ^27^ ^]^ However, we can infer that both *W*_F_ and VBM of the 2D/3D mixture lie between 2D and 3D perovskites. According to the UPS results, an energy band diagram of 3D, 2D/3D, 2D perovskites, and spiro‐OMeTAD is schematically illustrated in Figure 3D. The 2D/3D mixture resembles a buffer layer of energy bands, which bridges the VBM gap between the 3D and 2D perovskites. This improved energy level alignment facilitates hole transfer through the whole perovskite film and thereby decreases the charge–carrier recombination.^[^ ^6^ ^]^

Space charge limited current (SCLC) measurements are carried out to calculate the hole‐trap state density (*N*_t_) (Figure 3C).^[^ ^28^ ^]^ Hole‐only devices are prepared with a layout of ITO/PEDOT:PSS/perovskite/Spiro‐OMeTAD/Au without or with BTACl treatments for the test. The *N*_t_ can be determined via trap‐filled limit voltage (*V*_TFL_) using the following equation^[^ ^28^, ^29^, ^30^ ^]^

(1) $$N_{t}=\frac{2\varepsilon_{0}\varepsilon V_{\mathrm{TFL}}}{eL^{2}}$$

where *V*_TFL_ is the trap‐filled limit voltage, *ε*_0_ is the vacuum permittivity, *ε* is the relative dielectric constant, *e* is the elemental charge, and *L* is the thickness of perovskite films.^[^ ^31^ ^]^ The *V*_TFL_s of 0.74 and 0.38 V are obtained for the control and BTACl‐treated devices, which correspond to *N*_t_ of 2.74 × 10^16^ and 1.41 × 10^16^ cm^−3^, respectively. The trap‐state density of the BTACl‐treated film reduced by ~49% compared to the control sample.^[^ ^32^, ^33^ ^]^ It was reported that iodide vacancies (V_I_) were the dominant defects at the perovskite surface as they had the lowest formation energy than other usual defects.^[^ ^34^ ^]^ Therefore, we select V_I_ as an indicator to clarify the reduction of the trap‐state density. Via calculation, formation energies of the iodine‐related defects are determined to be −97.4 and −91.6 kJ mol^−1^ for the 3D MAPbI_3_ and 2D perovskites with *n* = 1 form, respectively (Figure S6, Supporting Information). This result explains that BTACl can suppress the generation of V_I_ to some extent.

The influence of BTACl treatment on optoelectronic performance of the perovskite films is studied via UV–Vis absorption, photoluminescence (PL), and time‐resolved PL (TRPL) measurements. Figure 3E demonstrates UV–Vis absorption spectra, in which a slight blue shift of the absorption onset is observed in the BTACl‐treated film.^[^ ^33^ ^]^ The PL spectra in Figure 3F show that the control sample has a peak position of 783 nm, agreeing well with the value reported in literature.^[^ ^30^, ^35^ ^]^ The peak position is 776 nm for the BTACl‐modified film, showing a slight blue shift compared to the control sample, which is consistent with the UV–Vis result. This observation is due to the formation of 2D perovskites after BTACl treatment, which gives a larger bandgap than 3D MAPbI_3_. Moreover, an increased PL intensity indicates more charge carriers in excited states undergo radiative recombination, suggesting less trap density at the sample surface after BTACl treatments. This conclusion is further verified by the PL spectra of the samples with an addition of Spiro‐OMeTAD layers on top, in which a reduced PL intensity is visible in the BTACl‐treated film as a result of more efficient transfer of change carriers from the perovskite to HTM (Figure 3G). The lifetime of the photogenerated charge carriers is further analyzed with TRPL measurements (Figure 3H). A biexponential equation is employed to fit TRPL data and the obtained parameters is present in Table S1 (Supporting Information).^[^ ^32^ ^]^ The trap‐assisted and radiation recombination are linked to the fast decay lifetime (*τ*_1_) and slow decay lifetime (*τ*_2_), respectively. The control sample has a *τ*_1_ of 41.34 ns with a 52.3% amplitude ratio (*A*_1_) and a *τ*_2_ of 143.54 ns with a 47.7% amplitude ratio (*A*_2_), while the BTACl‐treated sample shows longer *τ*_1_ (79.27 ns) and *τ*_2_ (336.06 ns) but a smaller *A*_1_ (27.18%). The longer decay lifetimes are mainly attributed to a faster disassociation of charge carriers caused by gradient energy levels in the perovskite film and a slower capture rate of charge carriers by traps. In addition, the reduced *A*_1_ implies the contribution of trap‐assisted recombination decreases with the introduction of BTACl.

**Figure 4:**
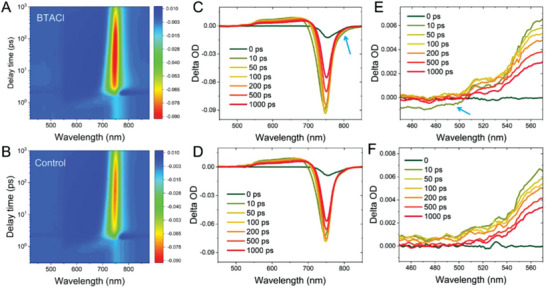
2D photon bleaching spectra in function of delay time for A) BTACl treated sample and B) the control sample. Selected temporal photon bleaching spectra for (C,E) the BTACl‐treated sample and (D,F) the control sample.

The transient absorption spectroscopy (TAS) measurements are used to study ultrafast charge carrier dynamics of the perovskite films with and without BTACl treatment. **Figure** **4**A,B illustrate the 2D images of the photon bleaching spectra of both perovskite samples as a function of delay times. The corresponding temporal spectra are displayed in Figure 4C,D and the selected spectral‐region are zoomed in and shown in Figure 4E,F. It is visible that the BTACl‐treated sample exhibits different bleaching features with the control sample specifically at ≈800 and 450–510 nm as indicated by the arrows (Figure 4C,E). In detail, the BTACl‐treated perovskite exhibits a distinct photon bleaching signal at a low energy state (≈800 nm) and it occurs only in *t* < 10 ps temporal region, which suggests that the energy transfer from 3D to 2D perovskites at their band‐edge is limited when the perovskites are both charged. The photon bleaching decay at the band‐edge of the 3D components of both perovskite samples are illustrated in Figure S7 (Supporting Information). The results indicate a faster carrier decay dynamic of the 2D/3D perovskite than the pure 3D perovskite in the range of *t* < 1000 ps. This is, we assume, in association with a higher carrier density as a result of the carrier injection from the additional 2D perovskite component of 2D/3D sample than the control sample. Notably, this fast carrier dynamics analysis (ps level) is based on total excited carrier population which includes the carriers responsible for the radiative recombination in TRPL (ns level).^[^ ^36^ ^]^

Moreover, it is noticed a distinct 2D photon bleaching signal at 510 nm for the BTACl‐treated sample (Figure 4E), which is assigned to the photon bleaching feature of *n* = 1 perovskites.^[^ ^37^ ^]^ Notably, very clear photon bleaching characters are observed at *t* = 10 ps in spectral wavelength < 510 nm, which implies that the 2D perovskite component introduces additional energy levels for collecting hot carriers. Additionally, this process occurs for only 10 ps, indicating that a potential transition channel for hot charge carriers cooling to lower energy states further assists the faster carrier decay at the band‐edge state as indicated in Figure S7 (Supporting Information). Also, the BTACl‐treated perovskite reveals a depressed feature of the excited state absorption (ESA) compared to the control sample at the same spectra region.^[^ ^37^ ^]^ These ESA states are normally associated with “halides‐to‐metal” charge transfer absorptions and might lead to unexpected Auger recombination due to the carrier accumulation.^[^ ^38^, ^39^ ^]^ We assume that the 2D perovskite component not only introduces additional energy states between the ground state and the higher energy state of the 3D perovskite for collecting correspondent hot carriers, but also suppresses the lead‐halide induced absorption at the interface of the 2D–3D heterojunction. As a result, the potential Auger recombination is suppressed, which contributes to enhanced device performance.^[^ ^40^, ^41^ ^]^

**Figure 5:**
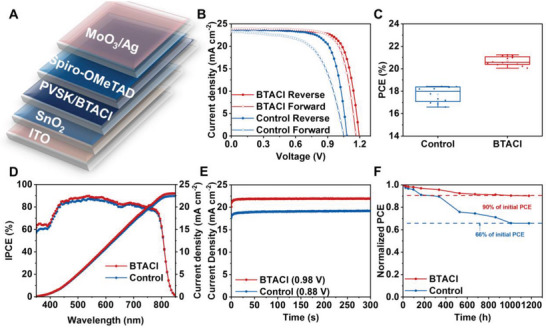
A) Schematic illustration of an implemented device. B) *J*–*V* cures of the best‐performing PSCs without and with BTACl treatments under reverse and forward scans. C) PCE distribution histogram (obtained from 12 independent devices), D) EQE spectra, and E) steady power output measurements for the control and BTACl‐treated PSCs. F) Normalized PCE of the nonencapsulated devices aged in a nitrogen glove box.

**Figure 6:**
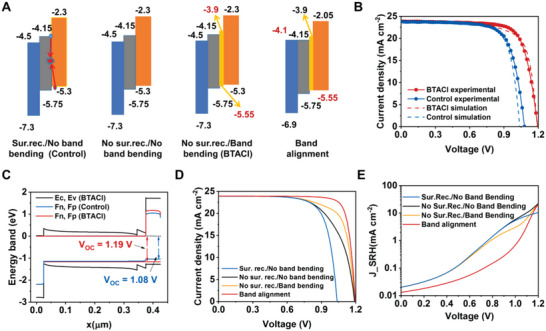
The device physics modeling analysis. A) The structures and band diagrams of semiconductor layers in the simulation. B) *J*–*V* curves obtained from SCAPS simulation in comparison with the experimental results for the cases of the control and BTACl‐treated devices. C) Band diagrams and the quasi‐Fermi levels for the cases of the control and BTACl‐treated films. D) *J*–*V* curves obtained from SCAPS simulation for all cases. E) Shockley–Read–Hall (SRH) recombination current density revealed by SCAPS simulation.

The perovskite films with and without BTACl treatments discussed above are individually used as the active layers for PSCs, and **Figure** **5**A shows the implemented device layout. The corresponding *J*–*V* characteristic curves of the best‐performing PSCs are compared in Figure 5B.^[^ ^42^ ^]^ The control PSCs are examined to have a PCE of 18.43% with a *V*_oc_ of 1.08 V, a short circuit current density (*J*_sc_) of 23.78 mA cm^−2^ and a fill factor (FF) of 71.74%, while the BTACl‐treated PSCs perform better with a PCE of 21.72%, a *V*_oc_ of 1.19 V, a *J*_sc_ of 23.85 mA cm^−2^, and an FF of 76.54%. Moreover, the BTACl‐treated device shows a smaller hysteresis than the control cell. The improvement in PCE is mainly attributed to the improved *V*_oc_ and FF. To interpret this occurrence, a self‐consistent physics device model simulation is performed. **Figure** **6**A illustrates the structures and band diagrams of semiconductor layers in the simulation. The control cell is represented by interfacial trap states recombination and the absence of band bending (Sur. rec./No band bending). And the BTACl‐modified device is signified by a band diagram with band bending and the absence of interface recombination (No sur. rec./Band bending). The trap states in the film are set based on the SCLC measurements in Figure 3C. We also simulate the cases of “No sur. rec./No band bending” and “Band alignment” for comparison.

Based on the parameters from the SCLC and UPS measurements, the model reproduces the *J*–*V* characteristics of the control and BTACl‐treated cells (Figure 6B and Table S2, Supporting Information). The key to the *V*_oc_ is the quasi‐Fermi level splitting at open circuit conditions, mainly affected by the recombination at the interface. As shown in Figure 6C, the simulation shows that the interface recombination is the main reason for the loss of quasi‐Fermi level splitting. By simply removing the interface recombination (No sur. rec./No band bending), the *V*_oc_ can increase to more than 1.19 V in this work. This indicates that the band bending has a minor effect on the *V*_oc_. A detailed view of the recombination and generation current density shows that interface recombination is the dominant recombination in the range of 1.0 to 1.2 V for the case of the control cell, while the Shockley–Read–Hall (SRH) recombination in the bulk is the main loss of performance for the BATCl‐treated cell (Figure S8, Supporting Information). The simulation results indicate that the reduction of trap states plays an essential role in increasing the *V*_oc_ of the devices in this work.

The energy‐band effect is analyzed by further simulating the cases of “No sur. rec./No band bending” and “Band alignment”. The introduction of a 2D perovskite layer with a highest occupied molecular orbital (HOMO) of −5.55 eV can increase the FF from 65.52% to 73.00% in devices without interfacial recombination (Figure 6D and Table S2, Supporting Information). Moreover, FF further increases to 82.61% in devices with energy band alignment between the transport layers and the perovskite layer (Figure 6D and Table S2, Supporting Information). Figure 6E shows that the SRH recombination related to bulk defects is significantly reduced in the case of solar cells with energy band optimization (No sur. rec./Band bending (BTACl) and Band alignment). The simulated results of the charge carrier density distribution and charge carrier recombination distribution also present diminished bulk effects (charge carrier density and SRH recombination) in the cases with energy band optimization (Figure S9, Supporting Information). The diminished bulk effects indicate a fast charge carrier extraction at the interface, which is enabled by the band bending and further enhanced by energy band alignment. A fast charge carrier extraction at the 2D interface revealed by the simulation of the band optimization cases is in line with the TAS results (Figure 4). It is worth noting that the simulation reveals an optimized device (“Band alignment”, the main loss mechanism retreats to the trap state in the bulk with a density of 1 × 10^16^ cm^3^) with the best performance of 23.63%.

Furthermore, we collect photovoltaic parameters out of 12 devices for each type individually. The average efficiency is 20.88 ± 0.16% for the BTACl‐treated device and 17.73 ± 0.49% for the control solar cell, respectively (Figure 5C). The related statistical plots of *V*_oc_, *J*_sc_, and FF are demonstrated in Figure S10 (Supporting Information). The smaller PCE deviation indicates a better repeatability for the device having 2D/3D perovskites. To verify the performance reliability, external quantum efficiency (EQE) spectra of both cells are probed and illustrated in Figure 5D. By integrating the photoresponse over the whole measured spectrum, the *J*_sc_is determined to be 22.44 and 23.02 mA cm^−2^ for the control and BTACl‐treated devices, respectively, which agree well with the values extracted from *J–V* sweeps. Moreover, the stabilized power output is tracked at the maximum power point with a stable *J*_sc_ of 19.12 mA cm^−2^ for the control device and 21.96 mA cm^−2^ for the BTACl‐treated cell (Figure 5E). It can be seen that both PSCs demonstrate excellent operational stability. The nonencapsulated BTACl‐treated PSC exhibits high stability as shown in the Figure 5F. The BTACl‐treated sample maintains 90% of its original PCE, while the control sample reduces to 66% of its initial efficiency after storage over 1000 h in the glove box. It is noticed that the water contact angle rises to 63.6° after the BTACl treatment (55.3° for the control film) (Figure S11, Supporting Information). The larger water contact angle indicates the improved hydrophobicity, which protects the perovskite from moisture penetration. To verify this inference, the control and BTACl‐treated films are exposed to various relative humidity (30%, 40%, and 50%) for different times for comparison (Figure S12, Supporting Information). The XRD curves show that MAPbI_3_ crystalline phase is preserved in both samples even after being stored in the 50% relative humidity for 17 d, suggesting that the methylammonium acetate (MAAc)‐derived perovskites have excellent stability against moisture. This observation is in line with our previous work.^[^ ^20^, ^35^ ^]^ However, the temporal evolution of XRD characteristics differs. Regardless of relative humidity, the peak intensities of (110) plane decrease over time in the control film, whereas they remain almost unchanged in the BTACl‐treated sample. This occurrence confirms that BTACl can improve environmental stability of perovskite films.

## 3 Conclusion

In the present work, we employ BTACl molecules to modify the perovskite‐film surface for high‐performance photovoltaic devices. This modification approach leads to the formation of a perovskite overlayer, which has a bilayer layout with an upper layer of pure 2D MAPbI_3_ of *n* = 1 phase and an under layer of 2D/3D mixed perovskites. This surficial low dimensional structure gives rise to a reduced trap density and an improved energy level alignment between the perovskite layer and HTL. As a result, a fast charge carrier transfer and a suppressed charge carrier recombination at the interface of 3D and low dimensional perovskites are observed. Benefiting from the low dimensional perovskites, the BTACl‐treated PSCs show improved *V*_oc_ and FF compared to the control devices. The simulation results clarify that the reduced charge carrier recombination and improved energy level alignment at the perovskite/HTL interface are the cause of *V*_oc_ and FF improvement, respectively.

The present work has deciphered the morphology of low dimensional perovskites induced by BTACl modification and elucidated the reason for the performance improvement of the BTACl‐treated PSC. The used research methods could provide a general guideline for further studies of film morphology and device physics of PSCs.

## 4 Experimental Section

### Materials

According to the procedure research, MAAc was prepared. Spiro‐OMeTAD and methylammonium iodide (MAI, 99.9%) were obtained from Advanced Election Technology Co., Ltd. Lead iodide (PbI_2_) and BTACl were purchased from TCL (Shanghai) Development Co., Ltd. Tin oxide hydrate (15% colloidal dispersion) was acquired from Alfa Aesar (China) Chemical Co., Ltd.

### Device Fabrication

ITO substrates were put into ethanol, detergent, deionized water, ethanol, and ultraviolet‐ozone (UVO) for 15 min in sequence. The tin oxide hydrate was mixed in deionized water with 1:5 vol prior to be deposited onto the cleaned ITO substrates with spin coating (3000 rpm, 30 s). After this step, the samples were annealed at 150 °C (30 min). Afterwards, the SnO_2_‐coated substrates were preheated for 2 min (95 °C). The perovskite solution was attained by mixing PbI_2_ (297.44 mg) and MAI (102.56 mg) in MAAc (1 mL), and was deposited onto the SnO_2_‐coated substrates by spin coating at 4000 rpm for 20 s then annealed at 100 °C (4 min). The BTACl (5 mg) was mixed in isopropanol (1 mL) to obtain the BTACl solution, which was deposited on the top of the as‐prepared perovskite films (3000 rmp, 30 s), and annealed at 100 °C (3.5 min). Afterwards, spiro‐OMeTAD, LiTFSI solution, and TBP were mixed in chlorobenzene (1 mL) to obtain the HTL solution. This solution was then deposited onto the perovskite films with spin coating (3000 rpm, 30 s) in the glove box. Finally, after a 20 h oxidation, MoO_3_ (5 nm) and Ag (100 nm) were thermally evaporated in sequence at a low pressure of 1 × 10^−4^ Pa.

### Film Characterizations

FLS1000 Edinburgh Instrument was used to get the TRPL and steady‐state PL data. Pulses were generated by 450 nm diode laser repetition rate diver. The samples were on glass substrates for PL measurements, while on glass/ITO/SnO_2_ substrates for TRPL measurements. The pump–probe TAS were measured by a lab‐established ultrafast spectral system in which an amplified Yb:KGW laser system (PHAROS, Light Conversion Ltd.) was used to generate synchronous laser beams (1030 nm with duration of 250 fs). An optical parametric amplifier (ORPHEUS, Light Conversion Ltd.) was employed to generate the 400 nm pump beam with a standard power density of 50 µJ cm^−2^. A YAG crystal was applied to generate continuously white light pulses (450–950 nm) acting as probe pulses. AFM measurement was carried out on the Dimension FastScan, Bruker at the tapping mode. And the SEM measurement was conducted with the 10 kV accelerating voltage and 6.0 mm working distance. The UV–Vis spectra were measured by U‐3900H UV–vis spectrophotometer. The GIWAXS data was carried out on the Synchrotron Radiation Facility BL02U2 beamline (Shanghai, China), operated at 10k eV X‐ray energy. The UPS characteristic recorded by the AXIS Ultra photoelectron spectrometer at a base pressure 5 × 10^−9^ mbar. Moreover, it was carried out with a sample bias of −8 V.

### Device Characterization

The *J*–*V* characteristic and steady‐state were recorded by a Enlitech SS‐F5‐3A solar simulator with a simulated AM1.5 100 mW cm^−2^ illumination from −0.2 to 1.4 V (a 0.01 V increment). The intensity of solar source was calibrated with a Enlitech standard Si photodiode. A 2.5 × 2 mm^2^ aperture size was selected as the illuminated area. The SCLC characteristic was conducted from 0 to 5 V under dark condition with the same way. PSCs were stored in a N_2_‐glove box at room temperature and with natural light exposure for long‐term stability test. Both water and oxygen content were less than 100 ppm.

### Device Simulation Analysis

The self‐consistent physics device model simulation was performed to further clarify the effect of reduced surface trap density and energy band bending induced by the BTACl modification. The simulation was implemented via SCAPS simulator, involving solution of Poisson, and drift‐diffusion equations with well‐handled boundaries and charge carrier recombination.^[^ ^43^, ^44^ ^]^ The problem and the architecture of the solar cell in the action panel of the SCAPS (Figure S13 and Table S3, Supporting Information), were defined. The perovskite/HTL interface was defined with interfacial trap states, which was modeled by the Pauwels–Vanhoutte theory.^[^ ^45^ ^]^ The energy band at the interface was adjusted by 2D perovskites with varied lowest unoccupied molecular orbital (LUMO) and highest occupied molecular orbital (HOMO).

### Statistical Analysis

In order to make a comparison, the TRPL data (Figure 3H) and PCE decay data (Figure 5F) were normalized to their respective initial values. The average PCEs were present as the format of mean ± standard deviation. Sample size (*n*) of PCE statistical analysis was shown in the corresponding figure legend. Statistical analysis of data was analyzed by Origin.

## Conflict of Interest

The authors declare no conflict of interest.

## Supporting information

Supporting Information

## Data Availability

Research data are not shared.
