# Supplementary material for: Deciphering the Morphology Change and Performance Enhancement for Perovskite Solar Cells Induced by Surface Modification
Source: Adv Sci (Weinh). 2022 Dec 1;10(3):2205342. doi: 10.1002/advs.202205342 (PMC9875650; doi:10.1002/advs.202205342)
Supplement: Supplementary file 1 — Supporting Information [file ADVS-10-2205342-s001.pdf]

Supporting Information

Deciphering the Morphology Change and Performance Enhancement for Perovskite Solar Cells Induced by Surface Modification

Nianci Guan, Yuezhou Zhang, Wei Chen, Zhengyan Jiang, Lei Gu, Ruixue Zhu, Deependra Yadav, Deli Li*, Baomin Xu, Leifeng Cao, Xingyu Gao, Yonghua Chen, Lin Song**

N. Guan, Y. Zhang, L. Gu, R. Zhu, D. Yadav, L. Song
Frontiers Science Center for Flexible Electronics, Xi'an Institute of Flexible Electronics (IFE), Northwestern Polytechnical University (NPU), Xi'an 710072, P. R. China.
E-mail: iamlsong@nwpu.edu.cn

D. Yadav
Pharmaceutical Sciences Laboratory, Faculty of Science and Engineering, Åbo Akademi University, FI-00520, Finland.

W. Chen, L. Cao,
Country College of Physics Engineering, Shenzhen Technology University (SZTU), Lantian Road 3002, Pingshan, Shenzhen 518118, China.
E-mail: chenwei@sztu.edu.cn;

Z. Jiang, B. Xu
Department of Materials Science and Engineering, Southern University of Science and Technology, Shenzhen 518055, China.

D. Li
Fujian Cross Strait Institute of Flexible Electronics (Future Technologies), Fujian Normal University, Fuzhou 350117, China.
E-mail: ifedlli@nwpu.edu.cn;

X. Gao
Country Shanghai Synchrotron Radiation Facility (SSRF), Zhangjiang Lab, Shanghai Advanced Research Institute, Chinese Academy of Sciences, Shanghai 201204, China.

Y. Chen
Key Laboratory of Flexible Electronics (KLOFE) & Institute of Advanced Materials (IAM),

Jiangsu National Synergistic Innovation Center for Advanced Materials (SICAM), Nanjing Tech University (NanjingTech), 30 South Puzhu Road, Nanjing 211816, Jiangsu, China.

*Corresponding author

E-mail address: chenwei@sztu.edu.cn;
ifedlli@nwpu.edu.cn;
iamlsong@nwpu.edu.cn;

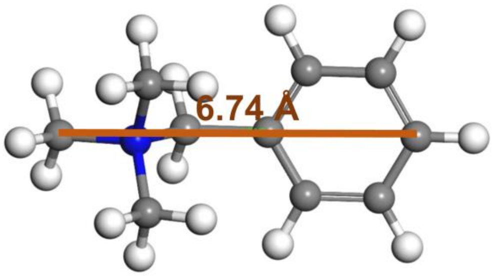

Figure S1. The size of a BTACl molecule.

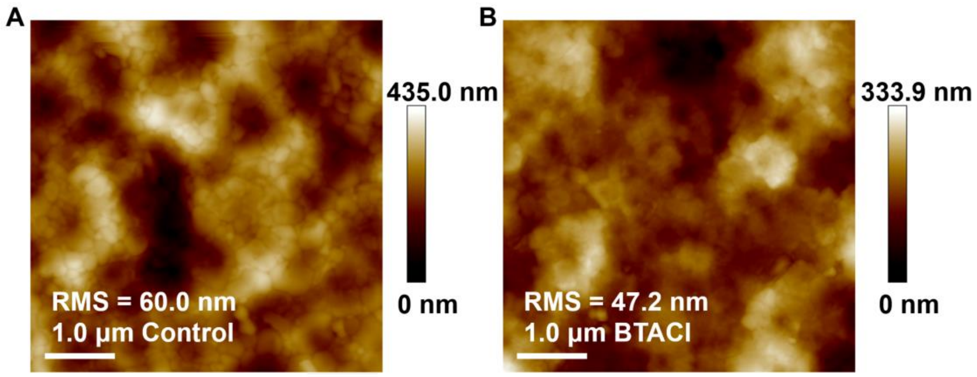

Figure S2. AFM images of the perovskite films (A) without and (B) with BTACl treatments.

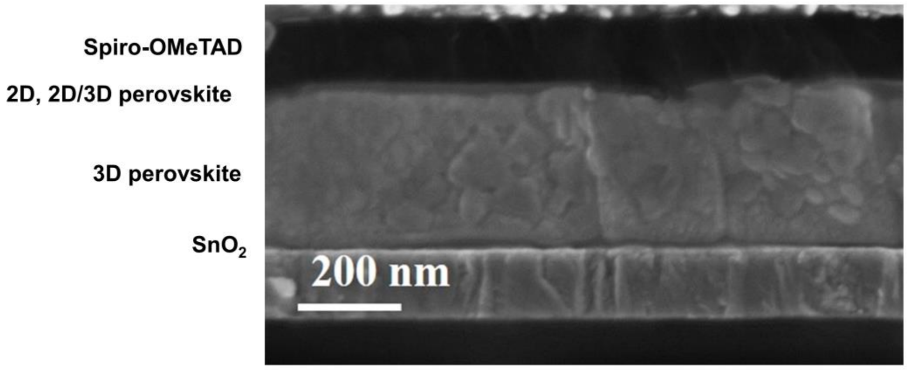

Figure S3. Cross sectional SEM image of a BTACl-treated PSC.

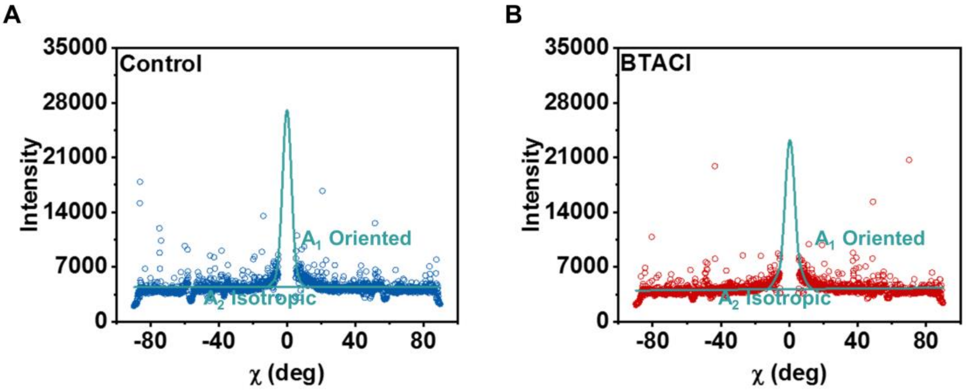

Figure S4. The tube integrals with an angular width q_r = 0.95 ~ 1.05 $Å^{-1}$ of (A) the control and (B) BTACl-treated perovskites.

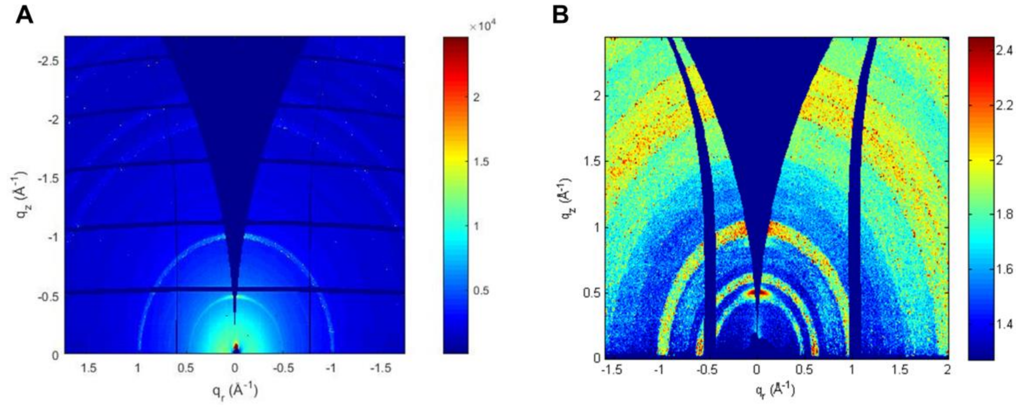

Figure S5. 2D GIWAXS data of the BTACl-treated perovskite film with an α_i of (A) 0.20° and (B) 0.21°.

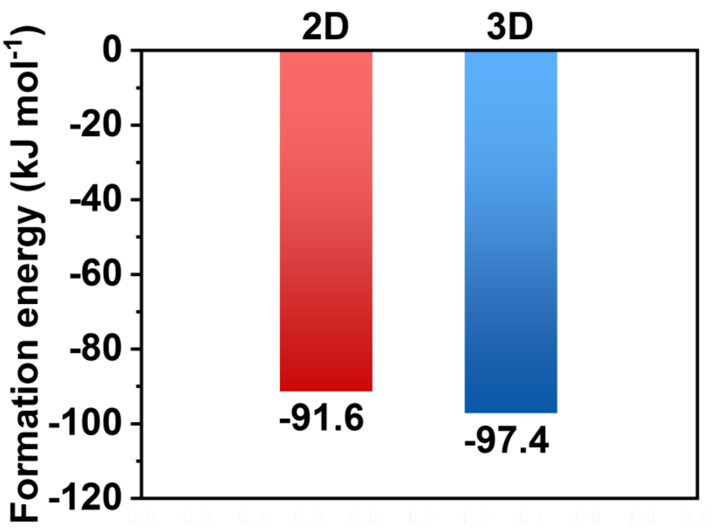

Figure S6. Formation energies of the iodide vacancies (V_I).

The formation energy of the iodine-related defect system is calculated using the CASTEP module of Materials Studio 8.0. The PbI_6 octahedra are set in the tetragonal phase of $MAPbI_3$, in which lattice constants are featured with a＝8.844 Å， b＝12.592 Å， c＝8.563 Å， α＝β＝γ＝90°. In this work, we constrcut supercell models of 10×10 for the BTACl-induced 2D perovskites and 10×10×10 for the 3D $MAPbI_3$. By removing an iodine atom individually, the model of PbI_6 with iodide vacancy (V_I) defects are obtained. The equation used to calculate the V_I formation energy in the rpesent work is

$$\Delta E = E(PbI_6)_{vacancy} - E(PbI_6) + \mu(X)$$

Where $E(PbI_6)_{vacancy}$ is the total energy of the defect system, $E(PbI_6)$ is the PbI_6 energy, and μ(X) is the chemical energy of element I. Through calculation, the V_I formation energies for 3D $MAPbI_3$ and 2D perovskites induced by BTACl can be determined, respectively. The results are shown in Figure S6, in which both formation energis are negative.

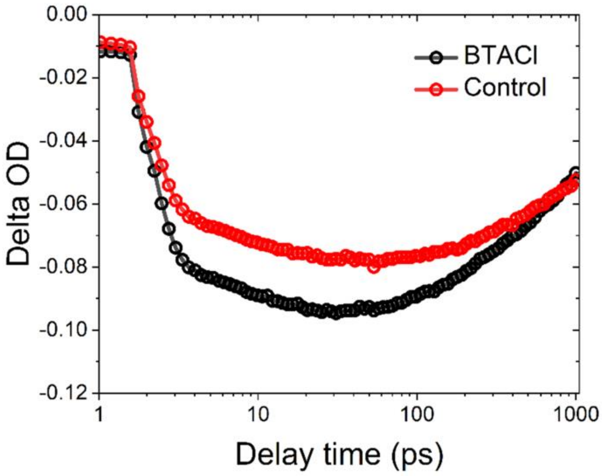

Figure S7. Charge carrier relaxation kinetics analysis at the band-edge of the perovskites.

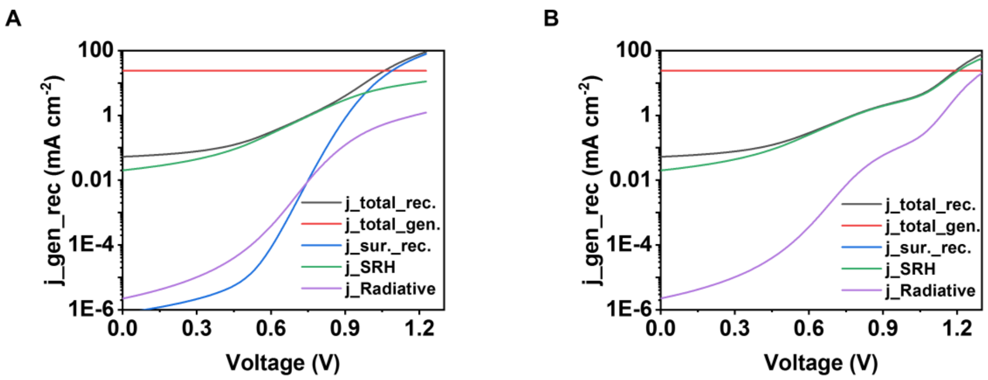

Figure S8. The detailed view of the recombination and generation current density for (A) the control and (B) BATCl-treated devices.

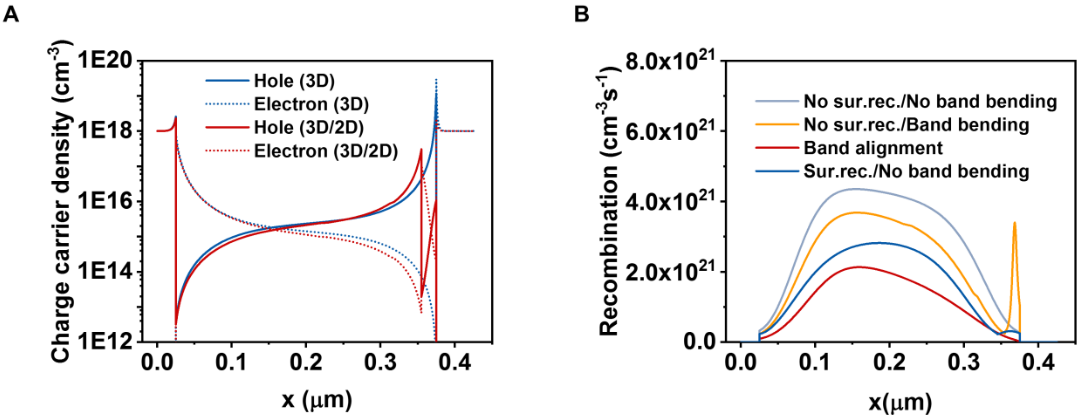

Figure S9. (A) The charge carrier density distribution of the cases of the control and BATCl-treated cells. (B) The recombination distributions of all studied cases.

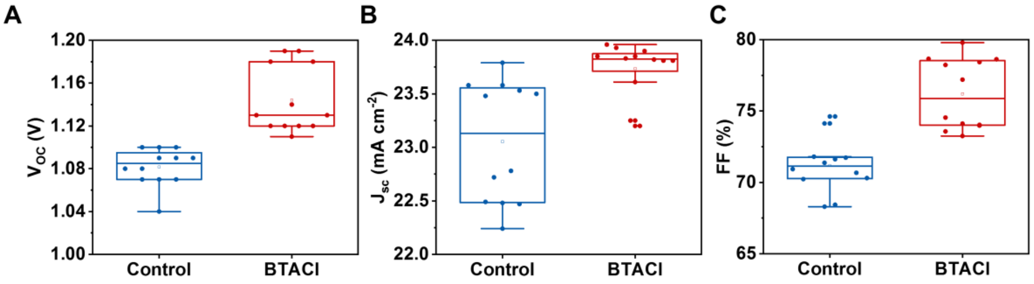

Figure S10. (A) V_{oc}, (B) J_{sc} and (C) FF distribution histograms for the PSCs without and with BTACl treatments.

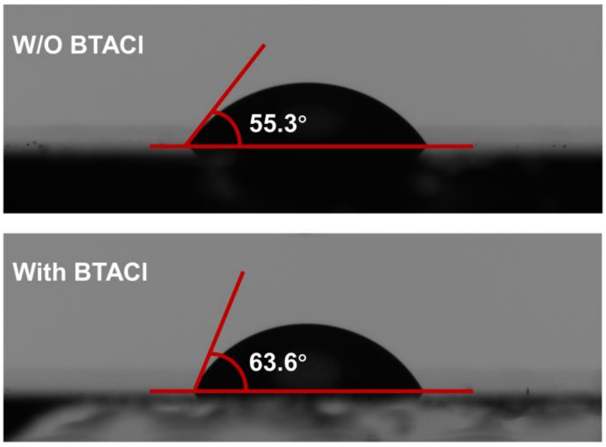

Figure S11. The water contact angle of (A) the control and (B) BTACl-treated samples.

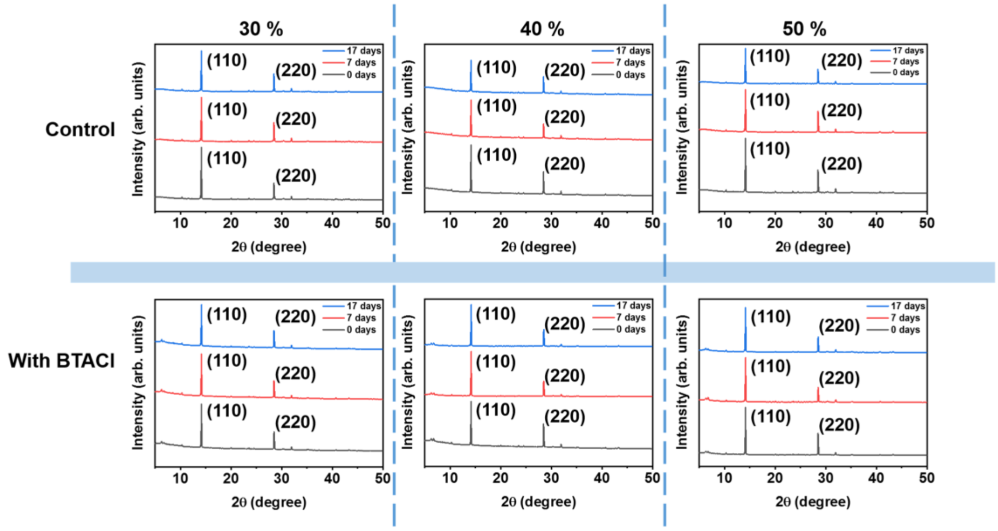

Figure S12. XRD spectra of the control and BTACl-treated perovskite films after exposure to various relative humidity (30 %, 40 % and 50 %) for different times.

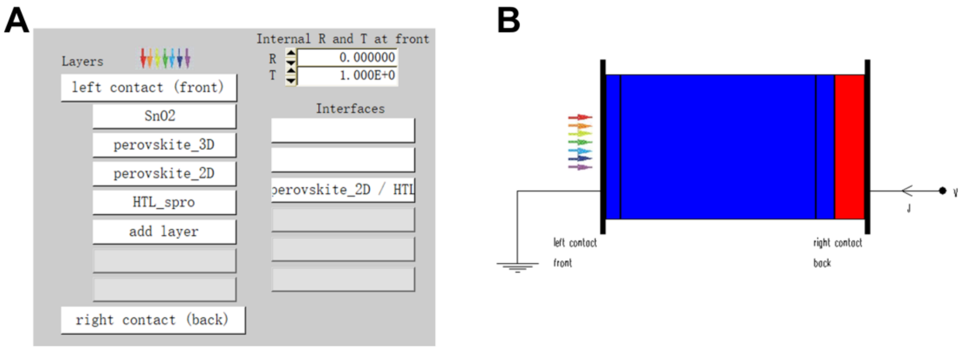

Figure S13. (A) The definition of the problem and (B) the architecture of the solar sell in the action panel of the SCAPS. The interface is defined between the perovskite layer and HTL with interface states which is modeled by the Pauwels-Vanhoutte theory. The energy band at the interface is adjusted by a perovskite_2D layer with varied HOMO and LUMO.

Table S1. TRPL parameters including time constant (τ_1 and τ_2) and amplitudes (A_1 and A_2) of the control and BTACl-treated perovskites.

	A_1 (%)	τ_1 (ns)	A_2 (%)	τ_2 (ns)	τ_{AV} (ns)
Control	52.27	41.34	47.73	143.54	124.99
BTACl	27.18	79.27	7.82	336.06	315.28

Table S2. The simulated photovoltaic parameters in comparison with the experimental results.

Perovskite solar cells	V_{OC} (V)	J_{SC} (mA cm^{-2})	FF (%)	PCE (%)
Sur. rec./No band bending (Control)	1.059	23.91	72.40	18.33
No sur. rec./Band bending (BATCl)	1.191	23.91	73.00	20.83
No sur. rec./No band bending	1.191	23.91	65.52	18.66
Band alignment	1.196	23.92	82.61	23.63
Experimental (Control)	1.08	23.78	71.74	18.43
Experimental (BATCl)	1.19	23.85	74.54	21.24

Table S3. SCAPS is a program to simulate the DC and AC electrical characteristics of thin film heterojunction solar cells. Version is SCAPS 3.3.07. Device parameters set in the simulation.

Parameter	ETL	3D	HTL	2D
Thickness(nm)	25	320/350	50	30/0
Bandgap (eV)	2.8	1.6	3.000	1.65
Electron affinity (eV)	(4.5/4.1)	4.15	2.450	3.85
Dielectric permittivity (relative)	9	6.500	3.000	6.500
CB effective density of states (1/cm^3)	2.200E+18	2.200E+18	2.200E+18	2.200E+18
VB effective density of states (1/cm^3)	1.000E+19	1.000E+19	1.000E+19	1.000E+19
Electron mobility (cm/Vs)	2.000E+2	2.000E+0	2.000E-4	2.000E+0
Hole mobility (cm/Vs)	2.000E+1	2.000E+0	1.000E-4	2.000E+0
Donor density ND (1/cm^3)	1.000E+18	1.000E+15	0	1.000E+15
Acceptor density NA (1/cm^3)	0.000E+0	0.000E+0	1.000E+18	0.000E+0
Radiative recombination coefficient (cm/s)	0.000E+0	1.000E-10	0.000E+0	1.000E-10
Auger electron capture coefficient (cm6/s)	0.000E+0	0	0.000E+0	0
Energy level with respect to Reference (eV)	1.5	0.600	1.450	0.600
Defect total (1/cm^3)	5.64+13	1.000E+16	1.000E+13	1.000E+16
